# Ambulation Mode Classification of Individuals with Transfemoral Amputation through A-Mode Sonomyography and Convolutional Neural Networks

**DOI:** 10.3390/s22239350

**Published:** 2022-12-01

**Authors:** Rosemarie Murray, Joel Mendez, Lukas Gabert, Nicholas P. Fey, Honghai Liu, Tommaso Lenzi

**Affiliations:** 1 Department of Mechanical Engineering, and Robotics Center, The University of Utah, Salt Lake City, UT 84112, USA; 2 Rocky Mountain Center for Occupational and Environmental Health, Salt Lake City, UT 84111, USA; 3 Walker Department of Mechanical Engineering, The University of Texas at Austin, Austin, TX 78712, USA; 4 State Key Laboratory of Robotics and Systems, Harbin Institute of Technology, Shenzhen 518055, China; 5 School of Computing, University of Portsmouth, Portsmouth PO1 3HE, UK

**Keywords:** A-mode ultrasound, ambulation mode classification, above-knee amputee, transfemoral amputee, lower-limb powered prosthesis, user intent recognition, sonomyography, neural signals

## Abstract

Many people struggle with mobility impairments due to lower limb amputations. To participate in society, they need to be able to walk on a wide variety of terrains, such as stairs, ramps, and level ground. Current lower limb powered prostheses require different control strategies for varying ambulation modes, and use data from mechanical sensors within the prosthesis to determine which ambulation mode the user is in. However, it can be challenging to distinguish between ambulation modes. Efforts have been made to improve classification accuracy by adding electromyography information, but this requires a large number of sensors, has a low signal-to-noise ratio, and cannot distinguish between superficial and deep muscle activations. An alternative sensing modality, A-mode ultrasound, can detect and distinguish between changes in superficial and deep muscles. It has also shown promising results in upper limb gesture classification. Despite these advantages, A-mode ultrasound has yet to be employed for lower limb activity classification. Here we show that A- mode ultrasound can classify ambulation mode with comparable, and in some cases, superior accuracy to mechanical sensing. In this study, seven transfemoral amputee subjects walked on an ambulation circuit while wearing A-mode ultrasound transducers, IMU sensors, and their passive prosthesis. The circuit consisted of sitting, standing, level-ground walking, ramp ascent, ramp descent, stair ascent, and stair descent, and a spatial–temporal convolutional network was trained to continuously classify these seven activities. Offline continuous classification with A-mode ultrasound alone was able to achieve an accuracy of 91.8±3.4%, compared with 93.8±3.0%, when using kinematic data alone. Combined kinematic and ultrasound produced 95.8±2.3% accuracy. This suggests that A-mode ultrasound provides additional useful information about the user’s gait beyond what is provided by mechanical sensors, and that it may be able to improve ambulation mode classification. By incorporating these sensors into powered prostheses, users may enjoy higher reliability for their prostheses, and more seamless transitions between ambulation modes.

## 1. Introduction

Limb loss affects 1.6 million people in the United States [1], and of these, 22% are transfemoral amputees [2]. In order to restore their ability to walk in the community, they need prostheses that allow them to walk on a wide variety of terrains, such as ramps, stairs, and level ground. Most available prostheses are passive devices and do not allow for activities that required net-positive energy, such as climbing stairs step-over-step [3]. Powered prostheses aim to address this limitation with battery-operated servomotors, sensors, and control. Current powered prostheses handle different types of terrain by using different ambulation-specific controllers. Therefore, to seamlessly transition between different terrain, the prosthesis must recognize the user’s intended ambulation task (e.g., walking, stairs climbing) [4]. Failure to recognize the user’s intended ambulation task can lead to gait instability or falls as the powered prosthesis may generate a behavior which is not compatible with the intended ambulation tasks, such as bending the prosthesis knee when it should be extended [5]. Thus, we need accurate ambulation-task predictions for powered prosthesis to become viable in real life.

Ambulation mode prediction is generally achieved using supervised machine learning [4]. Basically, a machine learning model is trained using a testing dataset that contains labelled data of one or more persons with amputation walking on different terrains and performing different ambulation tasks. Proper selection of sensors to embed in the prosthesis is critical to the success of this classification approach. Mechanical sensors, such as inertial measurement units (IMUs) or load cells [6,7,8,9,10,11,12,13,14,15,16] are commonly used in powered prostheses [17]. IMUs detect the acceleration and orientation of a segment of the prosthetic leg, while load cells detect the interaction force of the user on the prosthesis. Each of these sensors have inherent limitations, as IMUs can be affected by drift and walking speed, while load cells can struggle to classify mode during swing phase, when the forces and moments on the prosthesis are minimal [17]. Thus, additional sensor modalities are necessary to improve the accuracy of classification in powered prostheses.

Researchers have attempted to improve classification accuracy using sensors that monitor more than just the motion of the prosthesis or the physical interaction with the environment. For example, researchers have used sensors that monitor the environment such as range finder [18,19], 2D cameras [20], and depth cameras [21,22,23]. Moreover, researchers have proposed using sensors that can detect the muscle activations of the user such as force myography [24], surface electromyography (EMG) [25,26,27], and sonomyography [28]. EMG is the most common sensor modality used for this purpose. Although using EMG alone leads to lower accuracy than mechanical sensors alone, combining EMG with mechanical sensors can increase the classification accuracy [29,30,31]. Several studies have adopted the combined EMG/mechanical sensor approach [32,33,34,35,36,37,38,39,40,41,42,43,44]. However, EMG has intrinsic limitation such as the low signal-to-noise ratio [45], crosstalk in neighboring muscles [46], and the inability to distinguish between superficial and deep muscle activations [45]. Thus, classification accuracy may be improved by using a sensor modality that can provide a more reliable estimate of muscle activations.

An alternate approach to detecting muscle activity is muscle ultrasound, commonly known as sonomyography. Ultrasound transducers send a soundwave into the limb and measure the reflection back from different tissue layers. The propensity to reflect rather than transmit the sound (i.e., echogenicity) varies between different types of tissues. The recorded changes in the echogenicity pattern of the tissues reflect changes in the muscle shape. While electromyography and sonomyography both measure muscle activity, there are differences between them that may affect their suitability for neural control, specifically, ambulation mode recognition. Electromyography measures the electrical activity of the muscles. It increases with greater motor unit recruitment and faster firing rate of motor units. This could be expected to correlate with contractile muscle force. In contrast, sonomyography measures muscle deformation, which depends on both the joint angle and the contractile muscle force. In addition, muscle deformation can be influenced by pressure applied to the leg externally. Notably, sonomyography can differentiate between muscles at different depths and is unaffected by crosstalk from neighboring muscle activity. Thus, sonomyography can theoretically address some of the limitations identified with EMG.

Ultrasound sensors are available in different configurations. The most common type, B-mode ultrasound, uses an array of transducers to produce a two-dimensional image of the tissue cross-section. This sensing modality has been used for ambulation mode recognition in able-bodied subjects, and showed that sonomyography of the quadriceps with a linear discriminant analysis classification scheme can continuously classify ambulation mode better than surface EMG signals from muscles that span the hip, knee, and ankle [28]. However, these transducer arrays are inefficient, bulky, and difficult to integrate with the socket of a prosthesis in their current form factor, which limits the feasibility of using them for transfemoral amputees. In contrast, A-mode ultrasound uses a single transducer, and creates a one-dimensional image of the tissue. Although A-mode ultrasound provides much less comprehensive information about the muscle tissue, A-mode systems are substantially smaller and lighter, so they can easily be integrated with the socket. Thus, A-mode ultrasound has the potential to address some of the limitations of EMG without negatively affecting clinical viability.

A-mode ultrasound has shown promising results in upper-limb gesture classification among able-bodied subjects [47]. It can result in higher classification accuracy and is more robust to fatigue than EMG [48,49]. However, upper-limb gesture recognition involves classifying a static pose, rather than a cyclical movement as in ambulation mode recognition. In addition, the pressure applied by the socket of an upper-limb prosthetic varies less than a lower-limb prosthetic, which alternates between weight-bearing and non-weight-bearing throughout the gait cycle. Some of the changes in the ultrasound signal in the lower limb may be attributed to this significant change in pressure, rather than solely due to muscle contractions of the user. Thus, the viability of A-mode ultrasound for ambulation classification in lower-limb prostheses is unknown.

In this study, we provide the first demonstration of A-mode ultrasound for ambulation classification in individuals with transfemoral amputation. Specifically, we used four A-mode ultrasound sensors to continuously classify the ambulation mode of seven transfemoral amputee subjects walking in an ambulation circuit with level and inclined walkways and different sets of stairs. We use a spatial temporal convolutional network to classify level-ground walking, stair ascent, stair descent, ramp ascent, ramp descent, sitting, and standing. We compare the performance of sonomyography-based classification on different ambulation modes and explore the types of errors made by the classifier. We also compare the accuracy of sonomyography-based classification, IMU-based classification, and classification using a combination of sonomyography and IMU data. By providing the first demonstration of A-mode ultrasounds in transfemoral amputees, this study builds the foundation for future research aiming to improve classification accuracy in powered prostheses, a prerequisite for clinical viability.

## 2. Materials and Methods

Seven subjects with a transfemoral amputation participated in the study. Subject characteristics are shown in Table 1. The subject pool consisted of five men and two women, aged between 29 and 68 years old. Subjects were 1–22 years post amputation. Five subjects used a suction socket suspension while the remaining two used a lanyard style socket suspension. The study was conducted in accordance with the Declaration of Helsinki, and approved by the Institutional Review Board of The University of Utah (Protocol #00103197, approved 16 June 2021). Informed consent was obtained from all subjects involved in the study.

**Figure 1:**
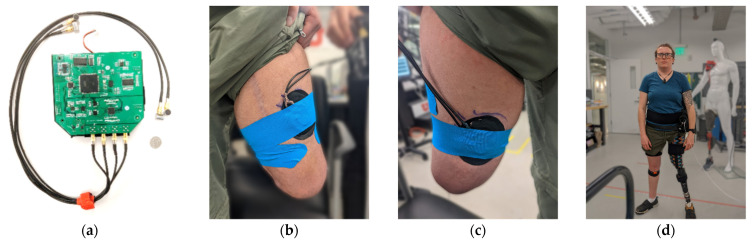
Sensor placement: (**a**) A-mode ultrasound system, with dime for scale. (**b**) Anterior US transducers placement. (**c**) Posterior US transducers placement. (**d**) Subject equipped with US system and IMU sensors.

The A-mode ultrasound system used in this study is shown in Figure 1a and consists of four transducers sampled sequentially at 80 Hz [50]. This portable ultrasound system was worn by the subject and transferred data to a laptop located on a desk via an ethernet cable. The ethernet cable was routed through an overhead track to avoid interference with the subject when they ambulated in the circuit. The ultrasound system was strapped to the subject’s waist, and the transducers were placed on the anterior and posterior side of the subject’s residual limb, as shown in Figure 1b,c. To place the sensors, the subject first removed their passive prosthesis and liner. They were then asked to flex and extend their hip on the amputation side. The approximate location of the rectus femoris and biceps femoris muscle bellies were identified through muscle palpation. Ultrasound gel and a pair of transducers were placed on each of these muscle bellies, and the ultrasounds signals were observed as the subject flexed and relaxed their muscle. The transducers were shifted slightly until a strong change in the signal was observed with muscle flexion. The transducers were then secured to the skin with kinesiology tape. The subject then placed their liner over the sensors and donned their socket and passive prosthesis. They were then instructed to walk around to check that the sensors did not interfere with socket suction or comfort. If needed, the sensor position was adjusted.

Walking kinematics were recorded with an inertia measurement unit (IMU)-based system (Xsens MVN, Enschede, Netherlands [51]). After the ultrasound transducers were in place, the IMUs were placed on the pelvis, thighs, shanks, and feet of the subjects (Figure 1d), and the system was calibrated. Data was recorded on a separate laptop at 80 Hz to match the ultrasound system recording rate. The ultrasound and motion capture data were synchronized by using a DAQ system (National Instruments USB-6001) to trigger the motion capture recording simultaneously with the ultrasound recording.

**Figure 2:**
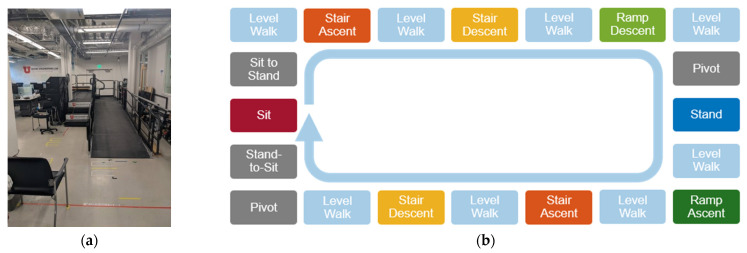
Experiment set up: (**a**) Ambulation circuit set up. (**b**) Ambulation mode sequence.

The subjects were then asked to complete 20 laps of the ambulation circuit, shown in Figure 2. The circuit began with the subject sitting quietly for 3 s. Subjects then stood and walked across level ground, ascended a flight of 4 stairs, and continued walking until having to turn and descend 2 stairs. Subjects then walked on level ground to reach a ramp, which they then descended. The ramp slope was 1:12 and the stair height was 7 inches. The ramp descent was followed by level-ground walking, which brought subjects to the mid-point of the circuit, where they were instructed to turn around and stand quietly for 3 s. After the quiet standing, subjects went through the reverse of the ambulation circuit. This involved level ground walking, followed by ramp ascent, level-ground walking, stair ascent of 2 stairs, level-ground walking, stair descent down 4 stairs, and level-ground walking up to the chair at the start. After reaching the chair, subjects turned around and sat down. The trial was completed once the subject was resting in a sitting position. While the subject walked on the circuit, an experimenter clicked a button at the transitions between different ambulation modes, which synced with the sonomyography program to record the ambulation mode. The ambulation modes recorded were: sitting, sit-to-stand, standing, stand-to-sit, pivot, level walking, stair ascent, stair descent, ramp ascent, and ramp descent.

### 2.1. Data Processing

Both the A-mode ultrasound data and the joint kinematics data were exported to and processed offline in MATLAB (Mathworks, Natick, MA, USA). At each time frame, the ultrasound system recorded a signal consisting of 997 sample points from one channel. The system iterated through the four channels, so that every fourth timeframe corresponded to the same ultrasound transducer being updated. The signal was zero-centered, rectified, and its envelope was calculated through a moving average convolution (sliding window of 77 sample points). Next, the signal was cropped to 960 sample points, with the 37 deepest datapoints being discarded. The signal was then segmented into 48 windows of 20 sample points. The mean of each window was calculated, resulting in 48 features for a single channel. This process is similar to upper-limb work described in [52]. The ultrasound data was then temporally segmented into overlapping windows of 1.25 s, or 100 time points. This was to capture the cyclical movement of the gait cycle, which can increase the steady-state accuracy of classification [15]. The windows overlapped by 99 time points, such that a classification would be made for every time frame. The final feature set consisted of 4 matrices of 100 rows and 48 columns. Each matrix represented one sensor, with the rows corresponding to the temporal dimension, and the columns corresponding to the spatial dimension. The label corresponds to the ambulation mode of the final timeframe. The window size of 100 timepoints was chosen through Bayesian optimization on a pilot data set. The results of this optimization are shown in Supplementary Figure S1.

The features selected from the IMU data were the position, velocity, acceleration, orientation, angular velocity, angular acceleration, sensor free acceleration, sensor magnetic field, and sensor orientation of the thigh, shank, and foot, as well as the direction, velocity and acceleration of the hip, knee, and angle, and the foot contacts on the amputation side, for a total of 113 pieces of information per time frame. This information was chosen as it represents the data typically available within a powered prosthesis. The data was similarly segmented into overlapping windows of 1.25 s, or 100 time frames. The label again corresponds to the ambulation mode of the final timeframe.

After the data was segmented into overlapping windows, datapoints labeled “sit-to-stand,” “stand-to-sit”, and “pivot” were discarded. This was due to the small number of datapoints containing these labels. The data from the 20 ambulation circuits were divided into training, testing, and validation datasets. Two laps of the ambulation circuit were reserved for the testing and validation of datasets, and the remaining laps were used for the training set. Validation and testing circuits were chosen as the middle two circuits to mitigate any potential effects of subject fatigue or sensor drift.

**Figure 3:**
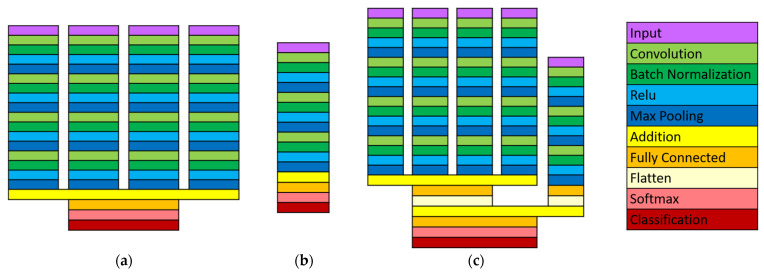
Classifier algorithm architecture: (**a**) Sonomyography spatial–temporal convolutional network. (**b**) Kinematic temporal convolutional network. (**c**) Combined sonomyography and kinematic network.

Three convolutional neural networks were designed to classify the ambulation mode using sonomyography data, kinematic data, or both. The network architectures are shown in Figure 3. The sonomyography classifier has a four-branch structure, with the signal from each of the four sensors as an input to a branch. Each branch consists of four blocks, that each contains a two-dimensional convolutional layer, a batch normalization layer, a ReLU activation layer, and a max pooling layer. Finally, the outputs of the four branches are added together and sent through a fully connected layer, a SoftMax layer, and a classification layer. The kinematic classifier has one branch, which receives all the kinematic data as an input. It has a similar structure of repeating blocks, but the convolutional layer is one-dimensional (along the temporal dimension). The combined data network structure takes the structure of the previous two networks and combines them. The complexity of the sonomyography classifier is 332 MFLOPS. The kinematic classifier has a complexity of 141 MFLOPS. The combined classifier has a complexity of 473 MFLOPS.

The convolutional neural network approach was chosen to take advantage of the relationship between data points that are adjacent spatially or temporally. It also produced better initial results on a single-subject pilot data set than a support vector machine, linear discriminant analysis, logistic regression, k-nearest neighbor, decision trees, naïve Bayes, or fully connected neural network classifier. The network structures for the sonomyography data and the kinematic data were chosen through testing on data collected from the pilot test. The number of repeated blocks, convolutional window size and stride length, and the pool size and stride length were selected through Bayesian optimization to provide the highest accuracy on that data set. The pilot data used to design the networks was not included in this study.

### 2.2. Outcome Measures

The accuracy, precision, recall, and F1 scores were calculated for each subject, and the mean and standard deviation of the group are reported. The accuracy for the entire dataset was defined as

(1) $$accuracy=\frac{correctly\;classified\;datapoints}{total\;datapoints}$$

The precision of each ambulation mode was defined as

(2) $$precision_{i}=\frac{datapoints\;correctly\;classified\;as\;mode_{i}}{total\;datapoints\;classified\;as\;mode_{i}}$$

The recall of each ambulation mode was defined as

(3) $$recall_{i}=\frac{datapoints\;correctly\;classified\;as\;mode_{i}}{total\;datapoints\;belonging\;to\;mode_{i}}$$

The F1 score of each ambulation mode was defined as

(4) $$F1\;score_{i}=2\times \frac{precision_{i}\times recall_{i}}{precision_{i}+recall_{i}}$$

The confusion matrix of the true and predicted ambulation modes for each timeframe in the test dataset was generated for each subject. It was normalized by dividing by the total number of datapoints in the test dataset. The group confusion matrix was calculated by taking the mean of the normalized confusion matrices and multiplying it by the mean number of datapoints in the test dataset.

For error analysis, consecutive misclassifications were grouped together into error segments. The duration of the error segment was calculated as the total time that the classifier was in an error state. For example, each timeframe is 12.5 ms apart, so four consecutive misclassified timeframes would be considered a single error segment with a duration of 50 ms. The time to transition of the error segment was calculated as the shortest time between any of the datapoints in the error segment, and the closest transition. For example, if an error segment began 2.5 s after a mode transition, and ended 50 ms before the next mode transition, the time to transition would be 50 ms. Finally, the errors were labeled either transition timing errors or steady state errors. Transition timing errors occurred at the boundary of an ambulation mode change, and indicate the classifier identified the correct mode change, but somewhat earlier or later than the mode change actually occurred. Steady state error segments occurred away from transitions and indicate that the classifier identified a mode change where none occurred.

Paired *t*-tests were performed to test for differences between the accuracy of the sonomyography-, kinematic-, and combined-classifiers on the test dataset. The Bonferroni–Holm correction for multiple comparisons was applied.

## 3. Results

### 3.1. Overall Performance

**Figure 4:**
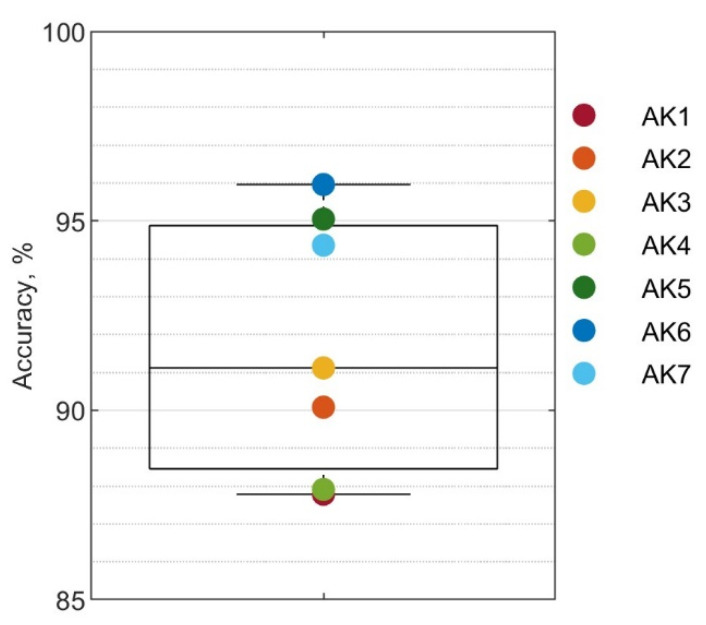
Overall accuracy of sonomyography classifier.

The mean classification accuracy using sonomyography alone was 91.8±3.4%. The distribution across subjects is shown in Figure 4 and ranged from 87.8% to 96.0%.

### 3.2. Performance by Ambulation Mode

**Figure 5:**
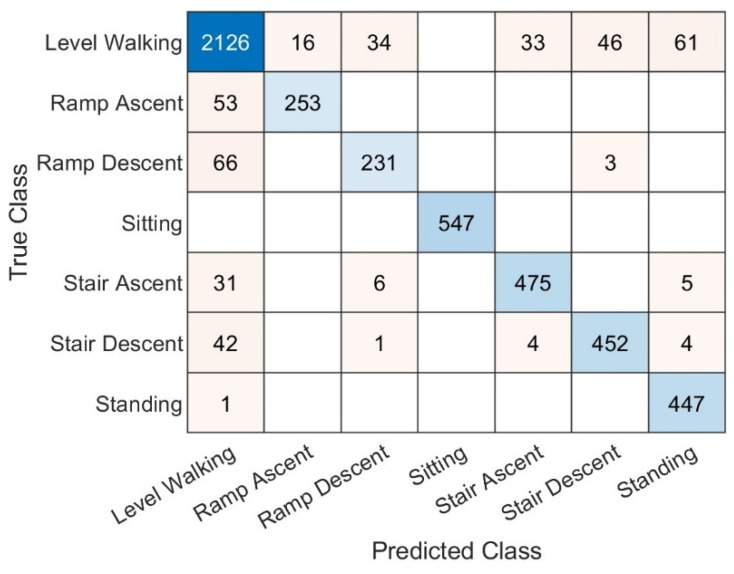
Combined confusion matrix for classification with sonomyography data alone. Correct classifications are shaded in blue, while incorrect classifications are shaded in pink. The depth of shading corresponds to the number of observations in each cell of the matrix as a proportion of the total number of observations.

The combined confusion matrix for all subjects using only sonomyography data is shown in Figure 5. Most confusion happened between level walking and other modes of ambulation. In particular, 17.3% of ramp ascent datapoints were classified as level walking, as well as 22.2% of ramp descent. Aside from level walking, ramp descent was confused with stair descent 1.0% of the time. Stair ascent was classed as ramp descent 1.1% of the time and standing 1.0% of the time. Finally, stair descent was classified as ramp descent 0.2% of the time, as stair ascent 0.8% of the time, and standing 0.8% of the time.

The precision, recall, and F1 score of each ambulation mode were calculated for each subject and the mean and standard deviation of the results for the group are shown in Table 2. Sitting was identified with 100% precision and recall. After sitting, ramp ascent had the highest precision (93.9 ± 3.8%) and standing had the highest recall (99.7±0.9%). Ramp descent had the lowest recall and precision at 86.0 ± 11.7% and 76.4 ± 21.5% respectively.

### 3.3. Error Types and Duration

**Figure 6:**
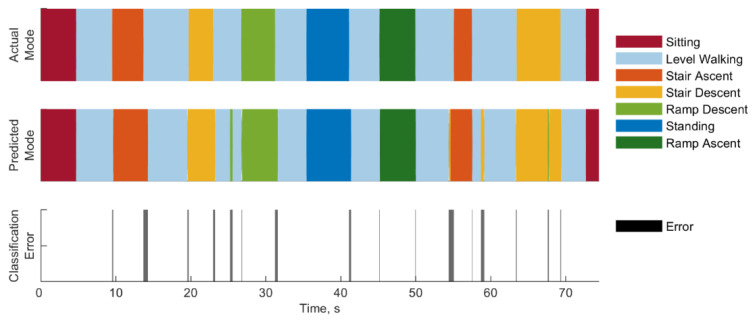
Classification errors for a representative subject, AK5.

The timing of errors of a representative subject (AK5) is shown in Figure 6. Most errors occur at transitions between different modes, with the classifier predicting this transition slightly early or late. This was most noticeable on transitions from stair ascent to level walking, ramp descent to level walking, and standing to level walking. Other errors occurred outside of transitions, such as the segments of level walking that were classified as ramp descent or stair descent, and the segment of stair descent which was classified as ramp descent.

**Figure 7:**
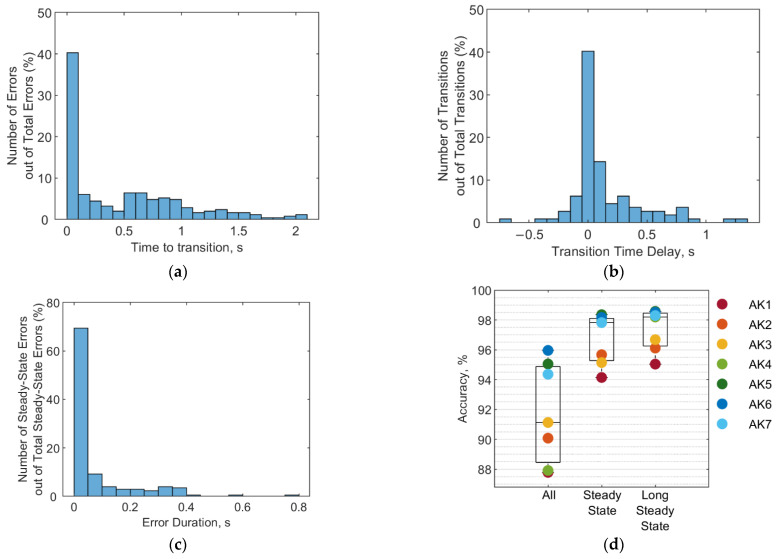
Error timing and duration: (**a**) Timing of errors relative to transitions among all subjects. (**b**) Distribution of transition timing delays among all subjects. (**c**) Distribution of error duration for steady state errors (those not associated with transitions). (**d**) Sonomyography classifier accuracy for three error definitions: all misclassified datapoints considered errors, only misclassified datapoints occurring during steady state considered errors, and only misclassifications occurring during steady state and of greater than 50 ms considered errors. The median, interquartile range, and total range for the group are shown in the boxplot. The colored points show individual results.

As with the representative subject, transitions between modes were associated with many of the classification errors throughout the entire subject pool. To further illustrate the influence of these transitions, Figure 7 shows the time between an error and its closest transition for the entire subject pool. Forty percent of errors began or ended less than 100 ms from a mode transition. Within this subset, 95% of errors began or ended precisely at an ambulation transition and could be described as discrepancies in timing of the transition between modes.

This variation in transition timing is shown in Figure 7b. The transition time delay was calculated as the difference between when the classifier identified the mode change and when the actual mode changed. Transition timing ranged from 662.5 ms early to 1262.5 ms late. Transitions were more likely to be recognized late rather than early, with 56% of transitions being recognized late, 21% recognized early, and 23% recognized precisely at the actual transition. Forty percent of transitions are recognized within 50 ms of the actual transition, and 61% within 150 ms of the actual transition.

Other errors were not associated with transitions. Transition errors were defined as those which occurred when the classifier identified the correct mode change, but with incorrect timing. The remaining errors were considered steady state and included cases where the classifier identified a mode change where one did not actually occur. For example, in Figure 6, at approximately 25 s into the trial, the classifier incorrectly identifies a segment of level walking as ramp descent. This would be considered a steady state error. The duration of these steady state errors among all subjects is shown in Figure 7c and ranged from 12.5 ms to 775 ms. Most errors (69.3%) lasted less than 50 ms, and 78.6% lasted less than 100 ms.

While one would ideally like to eliminate all errors, some errors may be more likely to impact performance than others. Transitioning slightly early or late may not impact a user as much as a steady state error. Even within steady state errors, those that are swiftly corrected may be ignored by the controller. Figure 7d shows the accuracy of the classifier for three different definitions of error. The first boxplot is the same as in Figure 4 and considers any misclassified datapoint an error. The second boxplot considers only steady state errors as true errors, and the third boxplot considers only steady state errors lasting more than 50 ms to be true errors. The accuracy for the three cases is 91.8±3.4%, 96.7±1.7%, and 97.3±1.4%, respectively.

### 3.4. Performance Comparison

**Figure 8:**
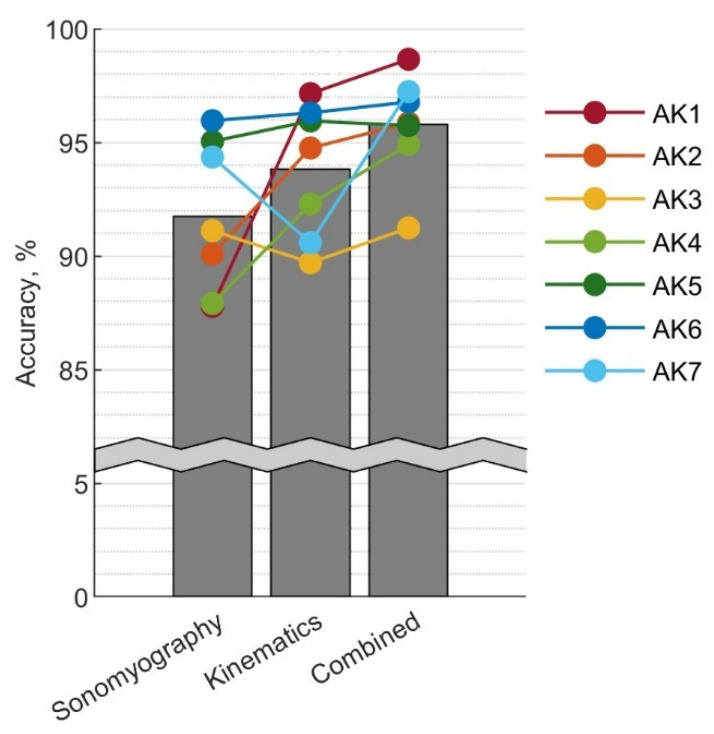
Classification accuracy when using sonomyogaphy data, kinematics data, or combined data from both sensors. The bars indicate the mean accuracy for the group, while individual subjects’ accuracies are shown in color. The grey waved line indicates a break in the y-axis.

The sonomyography classifier was compared with the kinematic classifier and combined data classifier. The overall accuracy of the three classifiers is shown in Figure 8. With sonomyography alone, the classification accuracy was 91.8±3.4%. Kinematics alone resulted in an accuracy of 93.8±3.0%, and the combined data classifier had an accuracy of 95.8±2.3%. No significant differences were observed between accuracies of the three classifiers.

Examining the performance of individual subjects reveals that the relative performance of the sonomyography and kinematic classifier depended on the subject. While some subjects attained greater accuracy with kinematics alone than sonomyography alone, for subjects AK3 and AK7 sonomyography was more accurate than kinematics. With the sole exception of AK5, subjects were able to achieve the greatest accuracy with the combined data set.

## 4. Discussion

A-mode ultrasound is a promising sensing modality for user intent detection, as it can encode information on muscle activations with high signal-to-noise ratio and distinguish between changes in superficial and deep muscles. The sensors are small and could be worn comfortably under the socket by seven transfemoral amputee subjects. Using a spatial–temporal convolutional network, sonomyography data alone could classify seven different ambulation mode with 91.8% accuracy.

Most misclassifications happened between level walking and other ambulation modes. A large portion of the errors occurred during transitions between modes. There are several possible reasons for this. Biomechanically, the transition between ambulation modes is not a discrete event, but a gradual one. Second, the mode transitions were recorded by an experimenter clicking the counter while the subject walked through the circuit. Human variability and reflexes could result in some inconsistency in the data labels. Finally, the temporal convolutional network structure may also have played a role. To capture the cyclical nature of ambulation, the data fed to the classifier contained information from the previous 100 timesteps. This may also cause the classifier to be slightly biased toward the previous mode rather than the next one, which would contribute to the transitions being identified late more often than early. A different network structure with more weight on the most recent timesteps may perform better on these transitions.

The steady state errors made by the classifier may pose a greater challenge to adaptation to online control than the transition errors. However, most of these errors were very brief. Studies have shown that ambulation mode transitions can be delayed by up to 90 ms without negative effects on the usability of the powered prosthesis [9]. A control strategy that required the sonomyography classifier to identify the new mode for four consecutive timesteps before switching could eliminate 69% of these steady state errors, while only delaying transitions by 50 ms. There still remain some longer duration steady state errors. Further work on ultrasound sensor placement and classification algorithm design may be able to mitigate these errors in the future.

Our results suggest that sonomyography alone provides similar classification accuracy to kinematic data alone. However, there are important limitations to this comparison, in that there are many more possible combinations of sensor placement and classification algorithms that could be explored, and further studies would be needed to illuminate the relative strengths of the sensing modalities. However, this initial comparison suggests that sonomyography data is comparable to IMU data as a source for ambulation classification.

Despite the similarity of the group mean accuracy for the sonomyography and kinematic classifiers, individual subjects did not necessarily achieve similar accuracy levels with the two classifiers. The subject-specific results in Figure 8 show that the relative accuracy of sensing modalities, for the given implementation of the classifiers, depended on the subject. There are a few possible causes for this. Some subjects may be better suited to sonomyography than others. This could be due to physiology, as some subjects had more obvious muscle bellies, while others had more subcutaneous tissue. Alternatively, it may be that the sensor placement for certain subjects was more informative than others. For some subjects, placing the sensor on the center of the muscle belly caused discomfort or disrupted suction when the socket was added, so the sensor had to be placed slightly off center. Finally, the two subjects that performed better with sonomyography than kinematics were both the only women in the group, as well as the only two that used lanyard style sockets. This suggests that gender differences in subcutaneous tissue and musculature in the thighs may play a role, or that suction socket types interfered with the sensor in a way that the lanyard style sockets did not. Further study on these effects could illuminate subpopulations that may particularly benefit from this sensing modality, or suggest better strategies for sensor-socket integration.

Regardless of which sensing modality was more accurate for a given subject, the combined data classifier almost always improved accuracy over either modality alone. This suggests that the sonomyography data may be capturing a slightly different aspect of the gait cycle than the IMU data, and that the combined dataset is more information rich as a result. A-mode ultrasound sensing may be a valuable addition to mechanical sensors, even in subjects where it is not more accurate than kinematic data alone.

Sonomyography also performs well in comparison to other neural signal classifiers available in the literature. A table of ambulation classification strategies intended for knee-ankle prostheses, adapted from [17], is shown in the Supplementary Table S1. Almost all studies employing EMG also incorporated mechanical sensors. In [29,30], EMG alone was compared to mechanical sensors alone, as well as the two combined. In [29], the mechanical sensors used were kinetic sensors: a 6 degree of freedom load cell in the prosthetic pylon, and pressure sensors in the soles of the shoes. The EMG classifier in that study was more accurate than the mechanical sensor classifier, particularly in swing phase. By contrast, in [30], the mechanical sensors included kinematic and inertial sensors as well. In this study, EMG alone was found to classify ambulation modes in transfemoral amputees with 6% error on steady state and 27% error on transitions, compared to 2% steady state and 20% transition error for mechanical sensors alone [30]. The combined data classifier had 1% error on steady state and 12% on transitions. While EMG alone was not better than the mechanical sensors, when combined, it was able to reduce the error by one percentage point on steady state and eight percentage points on transitions. In our study, sonomyography alone was able to classify steady state with 4.3% error, or 8.2% error on the entire dataset. The mechanical sensors were able to classify the total dataset with 6.2% error, and the combined classifier had 4.2% error. This suggests that the addition of sonomyography data to mechanical sensing data could reduce the error by two percentage points. These results suggest that A-mode ultrasound sonomyography performs better than EMG when used alone, and comparable when combined with mechanical sensors.

A previous work using B-mode ultrasound in able-bodied individuals has shown 99.8% classification accuracy [28], which is higher than this study with A-mode ultrasound. Although this result may be partly explained by the increased dimensionality of the data obtained using B-mode ultrasound, we believe that the main factor contributing to this difference is that the B-mode study analyzed able-bodied subjects, whereas our study focused on individuals with amputations using passive prostheses. Other differences in the protocol could have played a role. In the B-mode study, the transducer was placed over the rectus femoris, vastus lateralis, and vastus intermedius. While the rectus femoris is involved in hip flexion as well as knee extension, the vastus lateralis and vastus intermedius are solely involved in knee extension. Therefore, the data captured by the sensors would likely relate to both knee flexion and hip extension. In contrast, in this study placed sensors on the rectus femoris and biceps femoris, and since the subjects were transfemoral amputees, the data captured by these sensors would be related to hip flexion and extension. In addition, the B-mode ultrasound study attempted to classify five modes of ambulation, whereas the current study predicts seven distinct modes of ambulation. Moreover, the ramp ascent, ramp descent, and level walking conditions were collected on a treadmill, which has reduced variability between strides compared to level-ground walking. The ramp angle used was 10° rather than the ADA-compliant 4.8° used in this study, which may make the ramp ambulation modes more distinct from the level-walking mode. Additional studies using B-mode ultrasound on transfemoral amputees may help elucidate the relative benefits of each sonomyography modality for this population.

## 5. Conclusions

A-mode ultrasound sonomyography is a promising sensing modality for predicting widely varying ambulation activities in transfemoral amputee subjects. Overall, data from A-mode ultrasound can classify ambulation mode with comparable accuracy to kinematic data, with 91.8±3.4% and 93.8±3.0% accuracy, respectively. When combined, the accuracy may be improved over either data type alone, to 95.8±2.3%. This suggests that the information captured by sonomyography is different than the information captured by the kinematic data. Rather than being redundant, the combined dataset is richer than either one alone. A-mode sonomyography also performs comparably to other neural-signal-based ambulation classifiers reported in literature. This sensing modality may be a useful tool in reducing ambulation mode classification errors and the associated risks of gait instability and falls in transfemoral amputees.

## Figures and Tables

**Table 1:** Subject information.

Subject	Age	Years since Amputation	Socket Suspension	Sex	Weight (kg)	Height (m)
AK1	29	8	Suction	Male	65	1.78
AK2	68	5	Suction	Male	70	1.70
AK3	32	13	Lanyard	Female	59	1.60
AK4	32	4	Suction	Male	77	1.80
AK5	53	22	Suction	Male	100	1.93
AK6	54	2	Suction	Male	78	1.73
AK7	31	1	Lanyard	Female	59	1.68

**Table 2:** Sonomyography classifier mean performance by mode.

	Precision	Recall	F1
Level Walking	91.9 ± 5.9%	91.9 ± 1.7%	91.8 ± 3.2%
Ramp Ascent	93.9 ± 3.8%	82.4 ± 14.5%	87.2 ± 8.6%
Ramp Descent	86.0 ± 11.7%	76.4 ± 21.5%	79.4 ± 15.5%
Sitting	100±0%	100±0%	100±0%
Stair Ascent	92.5±5.2%	91.0±8.3%	91.5±5.0%
Stair Descent	90.8±9.4%	88.5±12.4%	89.1±8.5%
Standing	87.2±8.5%	99.7±0.9%	92.8±5.1%

## Data Availability

All data needed to support the conclusions of this manuscript are included in the main text or Supplementary Materials.

## Supplementary Materials

The following supporting information can be downloaded at: https://www.mdpi.com/article/10.3390/s22239350/s1, Figure S1. Classification accuracy versus temporal sequence length, and Table S1. Ambulation Mode Classification Strategies for Knee-Ankle Prostheses.
