# Supplementary material for: Ambulation Mode Classification of Individuals with Transfemoral Amputation through A-Mode Sonomyography and Convolutional Neural Networks
Source: Sensors (Basel). 2022 Dec 1;22(23):9350. doi: 10.3390/s22239350 (PMC9736589; doi:10.3390/s22239350)
Supplement: Supplementary file 1 [file sensors-22-09350-s001.zip › sensors-2041556-supplementary.pdf]

Supplementary Material

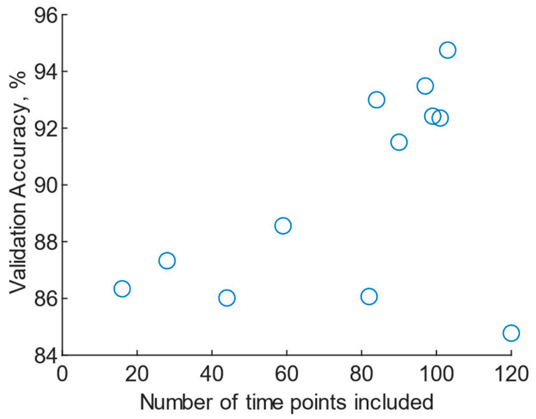

Figure S1: Classification accuracy versus temporal sequence length.

Table S1: Ambulation Mode Classification Strategies for Knee-Ankle Prostheses, based on tables provided in [18]. An extensive amount of work has been down on ambulation classification strategies for knee-ankle prostheses. Much of the work has focused on the integration of electromyography and mechanical sensors. A few of the studies have examined the relative contributions of each of these sensor types, and suggest that EMG may be an especially beneficial addition to mechanical sensors during swing phase. The limitations of the previous work include the limited number of activities: sitting and standing are often not considered, despite being important activities of daily living. Another limitation is that only 12 of the 33 studies shown included 5 or more transfemoral amputee subjects. Given the changes in musculature that accompany amputation, results obtained through EMG sensing in healthy subjects may not generalize to amputee subjects.

Article	Groups (N)[i]	Locomotion Activities[ii]	Sensors[iii]	Axes × Sensors	Offline/ Online	Recognition/ Prediction	Features[iv]	Algorithm[v]	Accuracy
Du et al. 2012 [36]	TF (9)	LW, SA, SD, RA, RD	EMG<br>Load cell	1 × 9<br>6 × 1	Off	P	MAV, SSC, WL, ZC<br>Mean, Max, Min	LDA	98
Du et al. 2013 [38]	TF (4)	LW, SA, SD, RA, RD	EMG<br>Load cell	1 × 7<br>6 × 1	Off	P	MAV, SSC, WL, ZC<br>Mean, Max, Min	EBA	92.5
Godiyal et al. 2018 [24]	TF (2)/ Healthy (8)	LW, SA, SD, RA, RD	FMG<br>Pressure	1 × 8<br>3 × 1	Off	R	Mean, Max, Min, SD, RMS, WL, SSC, MAD	LDA	96.1
Hernandez et al. 2012 [35]	TF (1)	LW, SA, SD, RA, RD, ST, SIT	Load cell<br>EMG	6 × 1<br>1 × 7	Off	R	MAV, SSC, WL, ZC<br>Mean, Max, Min	SVM	NP
Hernandez et al. 2013 [39]	Healthy (1)	LW, SA, ST	Load cell<br>EMG	6 × 1<br>1 × 7	Off and On	P	MAV, SSC, WL, ZC<br>Mean, Max, Min	SVM	99.9
Huang et al. 2009 [32]	TF (2)/ Healthy (8)	LW, SA, SD, OBS, Turns, ST	EMG<br>Pressure	1 × 11<br>2 × 1	Off	R	MAV, SSC, WL, ZC	LDA	95.5
Huang et al. 2010 [33]	TF (1)/ Healthy (5)	LW, SA, SD, OBS	EMG<br>Pressure	1 × 11<br>NP	Off	P	MAV, SSC, WL, ZC	LDA	NR
Huang et al. 2011 [29]	TF (5)	LW, SA, SD, RA, RD, OBS	EMG<br>Load cell<br>Pressure	1 × 11<br>6 × 1<br>NP	Off	P	MAV, SSC, WL, ZC<br>Mean, Max, Min	SVM	100
Liu et al. 2016 [19]	TF (1)/ Healthy (6)	LW, SA, SD, RA, RD	EMG<br>Load cell<br>IMU<br>Laser	1 × 8<br>6 × 1<br>6(A, G, α) × 1<br>1 × 1	Off and On	P	MAV, SSC, WL, ZC<br>Mean, Max, Min, SD	LDA	~98
			EMG	1 × 7		P	MAV, SSC, WL, ZC	EBA/LIFT	94.3

Liu et al. 2017 [44]	TF (2)/ Healthy (2)	LW, SA, SD, RA, RD	Load cell	6 × 1	Off and On		Mean, Max, Min, SD		
Liu et al. 2017 [8]	TF (2)/ Healthy (3)	LW, SA, SD, RA, RD	IMU<br>Pressure	4(A, G) × 1<br>2 × 2	Off	R	ICC	HMM	95.8
Rabe et al. 2021 [28]	Healthy (10)	LW, SA, SD, RA, RD	EMG<br>B-mode US	1 × 8<br>128 × 1	Off	R	MAV, SSC, ZC, WL, AR<br>Mean Intensity	LDA	99.8
Simon et al. 2017 [9]	TF (6)	LW, SA, SD, RA, RD, ST	Joint Angle<br>Joint Velocity<br>Motor Current<br>IMU<br>Load cell	1 × 2<br>1 × 2<br>1 × 2<br>6(A, G, α) × 1<br>6 × 1	Off	P	WT	DBN	99.6
Spanias et al. 2014 [41]	TF (4)	LW, SA, SD, RA, RD	Joint Angle<br>Joint Velocity<br>Motor Current<br>IMU<br>Load cell<br>EMG	1 × 2<br>1 × 2<br>1 × 2<br>6(A, G, α) × 1<br>1 × 1<br>1 × 9	Off	P and R	MAV, SSC, WL, ZC, AR<br>Mean, Max, Min, SD, IV, FV	LDA	~ 96
Spanias et al. 2015 [31]	TF (6)	LW, SA, SD, RA, RD	Joint Angle<br>Joint Velocity<br>Motor Current<br>IMU<br>Load cell<br>EMG	1 × 2<br>1 × 2<br>1 × 2<br>8(3A, 3G, 2α) × 1<br>6 × 1<br>1 × 4	Off	P	MAV, SSC, WL, ZC, AR<br>Mean, Max, Min, SD, IV, FV	DBN	~ 99
Spanias et al. 2016a [42]	TF (8)	LW, SA, SD, RA, RD	Joint Angle<br>Joint Velocity<br>Motor Current<br>IMU<br>Load cell<br>EMG	1 × 2<br>1 × 2<br>1 × 2<br>6(A, G, α) × 1<br>1 × 1<br>1 × 9	Off	P	MAV, SSC, WL, ZC, AR<br>Mean, Max, Min, SD	DBN	NR

Spanias et al. 2016b [13]	Healthy (2)	LW, SA, SD, RA, RD, ST	Joint Angle<br>Joint Velocity<br>Motor Current<br>IMU<br>Load cell	1 × 2<br>1 × 2<br>1 × 2<br>10(3A, 3G, 4α) × 1<br>6 × 1	Off and On	P and R	Mean, Max, Min, SD, IV, FV	DBN	96.7
Spanias et al. 2017 [14]	TF (3)	LW, SA, SD, RA, RD, ST	Joint Angle<br>Joint Velocity<br>Motor Current<br>IMU<br>Load cell	1 × 2<br>1 × 2<br>1 × 2<br>10(3A, 3G, 4α) × 1<br>6 × 1	Off and On	P and R	Mean, Max, Min, SD, IV, FV	DBN	98.8
Spanias et al. 2018 [43]	TF (8)	LW, SA, SD, RA, RD, ST	Joint Angle<br>Joint Velocity<br>Motor Current<br>IMU<br>Load cell<br>EMG	1 × 2<br>1 × 2<br>1 × 2<br>10(3A, 3G, 4α) × 1<br>6 × 1<br>1 × 8	Off and On	P and R	MAV, SSC, WL, ZC, AR<br>Mean, Max, Min, SD, IV, FV	DBN	95.97
Stolyarov et al. 2018 [10]	TF (6)	LW, SA, SD, RA, RD	IMU	6(A, G) × 1	Off	P	Mean, Max, Min, SD	LDA	94.1
Su et al. 2019 [11]	TF (1)/ Healthy (10)	LW, SA, SD, RA, RD	IMU	6(A, G) × 3	Off	R	Raw Data	CNN	89.2
Woodward et al. 2016 [6]	TF (6)	LW, SA, SD, RA, RD	IMU<br>Joint Angle<br>Joint Velocity<br>Joint Current<br>Load cell	7(3A, 3G, 1α) × 1<br>1 × 2<br>1 × 2<br>1 × 2<br>6 × 1	Off	P	Mean, Max, Min, SD, IV, FV	ANN	98.9
Young et al. 2013a [7]	TF (4)	LW, SA, SD, RA, RD	IMU<br>Joint Angle	6(A, G) × 1<br>1 × 2	Off	P	MAV, SSC, WL, ZC, AR	DBN	~ 98.2

			Joint Velocity	1 × 2					
			Joint Current	1 × 2			Mean, Max, Min, SD		
			Load cell	1 × 1					
			EMG	1 × 9					
Young et al. 2013b [15]	TF (6)	LW, SA, SD, RA, RD	IMU	6(A, G) × 1	Off	P	Mean, Max, Min, SD	DBN	~ 98
			Joint Angle	1 × 2					
			Joint Velocity	1 × 2					
			Joint Current	1 × 2					
			Load cell	1 × 1					
Young et al. 2013c [40]	TF (4)	LW, SA, SD, RA, RD	IMU	6(A, G) × 1	Off	P	MAV, SSC, WL, ZC, AR	LDA	86.4
			Joint Angle	1 × 2					
			Joint Velocity	1 × 2					
			Joint Current	1 × 2					
			Load cell	1 × 1			Mean, Max, Min, SD		
			EMG	1 × 7					
Young et al. 2014a [12]	TF (6)	LW, SA, SD, RA, RD	IMU	6(A, G) × 1	Off	P	Mean, Max, Min, SD	LDA	~ 99
			Joint Angle	1 × 2					
			Joint Velocity	1 × 2					
			Joint Current	1 × 2					
			Load cell	1 × 1					
Young et al. 2014b [30]	TF (8)	LW, SA, SD, RA, RD	IMU	6(A, G) × 1	Off	P	MAV, SSC, WL, ZC, AR	DBN	~ 99
			Joint Angle	1 × 2					
			Joint Velocity	1 × 2					
			Joint Current	1 × 2					
			Load cell	1 × 1			Mean, Max, Min, SD		
			EMG	1 × 9					
Young et al. 2016 [16]	TF (8)	LW, SA, SD, RA, RD	IMU	6(A, G) × 1	Off	P	Mean, Max, Min, SD, IV, FV	DBN	~ 99
			Joint Angle	1 × 2					
			Joint Velocity	1 × 2					
			Joint Current	1 × 2					
			Load cell	1 × 1					
Zhang et al. 2011 [34]	TF (1)/ Healthy (1)	LW, SA, SD, RA, RD	Load cell	6 × 1	Off and On	P	MAV, SSC, WL, ZC	LDA	> 97
			EMG	1 × 11			Mean, Max, Min		

Zhang et al. 2013 [37]	TF (4)	LW, SA, SD, RA, RD, ST, SIT	IMU<br>Load cell<br>EMG	6(A, G) × 2<br>6 × 1<br>1 × 8	Off and On	P	MAV, SSC, WL, ZC<br>Mean, Max, Min	SVM	95
Zhang et al. 2019a [22]	TF (3)/ Healthy (6)	LW, SA, SD, RA, RD	Depth Camera<br>IMU	224 × 171<br>3(α) × 1	Off	P	Raw data	CNN + HMM	96.4
Zhang et al. 2019b [23]	TF (3)/ Healthy (6)	LW, SA, SD, RA, RD	Depth Camera<br>IMU	224 × 171<br>3(α) × 1	Off	P	Raw data	CNN	94.9
Zhang et al. 2012 [18]	Healthy (1)	LW, SA, SD, ST	IMU<br>Laser<br>Load cell<br>EMG	6(A, G) × 1<br>1 × 1<br>6 × 1<br>1 × 7	Off and On	P	MAV, SSC, WL, ZC<br>Mean, Max, Min	LDA	97.6

[i] Groups consisted of healthy or transfemoral amputee (TF) subjects.

[ii] Locomotion activities consisted of level walking (LW), stair ascent (SA), stair descent (SD), ramp ascent (RA), ramp descent (RD), standing (ST), sitting (SIT), stepping over an obstacle (OBS), and turning.

[iii] Specific sensors included electromyography (EMG), force myography (FMG), inertial measurement units (IMU), pressure sensors, motor current, joint current, joint angles, joint velocities, laser range finders (Laser), load cells, and depth cameras.

[iv] Features included raw data, mean, maximum (Max), minimum (Min), mean absolute value (MAV), root mean square error (RMS), slope sign changes (SSC), waveform length (WL), zero crossings (ZC), standard deviation (SD), initial value (IV), final value (FV), autocorrelation coefficients of an autoregressive model (AR), and wavelet transform (WT).

[v] Algorithms included linear discriminant analysis (LDA), entropy based algorithm (EBA), support vector machine (SVM), dynamic Bayesian network (DBN), learning from testing data (LIFT), artificial neural network (ANN), convolutional neural network (CNN), and hidden Markov model (HMM).
